# Sulfur Polymer to Develop Low-Carbon Reclaimed Asphalt Pavements

**DOI:** 10.3390/polym18020168

**Published:** 2026-01-08

**Authors:** Mohammad Doroudgar, Mohammadjavad Kazemi, Shadi Saadeh, Mahour Parast, Elham H. Fini

**Affiliations:** 1 Department of Civil Engineering and Construction Engineering Management, California State University Long Beach, Long Beach, CA 90840, USA; shayan.doroudgar01@student.csulb.edu; 2 School of Sustainable Engineering and the Built Environment, Arizona State University, Tempe, AZ 85281, USA

**Keywords:** sulfur polymer, hot mix asphalt (HMA), low-carbon modifier, reclaimed asphalt pavement (RAP), sustainable pavement materials

## Abstract

The incorporation of reclaimed asphalt pavement (RAP) offers significant environmental benefits; however, its use is often limited by an increased susceptibility to cracking due to the insufficient elasticity of the severely aged RAP binder. This limitation is conventionally mitigated using polymers such as styrene–butadiene styrene, which, despite their effectiveness, are costly and carbon intensive. This paper introduces a low-carbon sulfur-based ternary polymer developed through TiO_2_-catalyzed inverse vulcanization of elemental sulfur to be used as a modifier to address the abovementioned challenge at the asphalt mixture level. The sulfur polymer containing waste cooking oil and metal-rich biochar was incorporated into hot-mix asphalt having 25% RAP. The mixture specimens were evaluated before and after accelerated thermal and ultraviolet aging. Cracking resistance was measured using the Indirect Tensile Asphalt Cracking Test (IDEAL-CT), while resistance to rutting and moisture damage were assessed through the Hamburg Wheel Tracking Test (HWT). IDEAL-CT findings showed improved ${CT}_{Index}$ values for the modified mixture under unaged conditions and after three days of thermal aging, with smaller variations noted after prolonged thermal aging and during the combined thermal–ultraviolet aging process. Results from the HWT test revealed that the addition of the sulfur polymer did not negatively impact resistance to rutting or moisture damage; all mixtures remained significantly below rutting failure thresholds. Furthermore, a simplified environmental analysis indicated that substituting 10 wt% of petroleum binder with the sulfur polymer lowered the binder’s cradle-to-gate global warming potential by around 11%. In summary, study results showed that the newly developed sulfur polymer system has the potential to improve cracking resistance even when exposed to select accelerated aging protocols while decreasing embodied carbon, thus endorsing its viability as a sustainable modifier for asphalt mixtures.

## 1. Introduction

The growing need for pavements that last longer and have a lower environmental impact has led to the increased use of reclaimed asphalt pavement (RAP) in contemporary asphalt mixtures as a sustainable and resource-efficient option [1,2,3]. Higher levels of RAP provide significant sustainability advantages by lowering the demand for virgin binders and aggregates while encouraging circular material usage [4,5,6]. Nevertheless, this environmental benefit presents a significant performance issue: the aged binder in RAP is notably oxidized and rigid, which raises the mixture’s modulus and greatly increases its vulnerability to cracking, especially in cooler temperatures [7,8,9,10]. As agencies and practitioners strive for greater RAP incorporation to fulfill sustainability objectives [11,12], finding a balance between material circularity and long-term mechanical resilience has become a primary design challenge [5,13]. Without effective modification techniques, mixtures with high RAP content may experience early cracking and shortened service life, thereby conflicting with the sustainability goals they aim to achieve [3,14,15].

Waste cooking oil (WCO) has been extensively researched as a rejuvenator that can restore the low-temperature and rheological characteristics of aged binder [16,17]. Previous studies indicate that WCO can reduce viscosity, enhance fatigue resistance, and improve low-temperature cracking performance in high-RAP systems [7,8,18]. These advantages make WCO an important tool for mitigating the excessive stiffness associated with aged RAP binders [8,19]. However, when used alone as a modifier, WCO mainly functions as a softening agent and does not contribute to the structural reinforcement of the asphalt matrix [7,10,20,21]. It has been observed that at high dosages of WCO, excessive softening, decreased high-temperature stability, and increased vulnerability to long-term oxidative aging can occur [9,10]. These drawbacks suggest that while WCO-based rejuvenation is essential for restoring flexibility, it is inadequate on its own to guarantee balanced performance and long-term durability in high-RAP mixtures. Therefore, WCO needs additional reinforcement strategies capable of re-establishing stiffness, managing aging, and maintaining mechanical integrity throughout its service life [22,23].

Sulfur and sulfur-based polymer networks present significant reinforcement potential. The process of inverse vulcanization using elemental sulfur along with bio-oils, especially when metal-oxide catalysts like TiO_2_ are present, results in high-sulfur polymers that exhibit increased viscosity and robust sulfur-organic cross-linking [24,25,26,27]. When these materials are added to bituminous systems, the polymerization of sulfur can enhance stiffness and viscoelastic recovery through the creation of S–C and S=C bonds, especially when paired with bio-modifiers that have unsaturated functional groups such as WCO [28,29]. However, sulfur-rich binders might be susceptible to surface oxidation when exposed to ultraviolet (UV) light if the network does not have adequate stabilization [30,31], which emphasizes the importance of UV-resistant additives [32,33].

Recent mechanistic research indicates that asphalt containing sulfur experiences quick photochemical oxidation, resulting in the formation of sulfoxide, sulfone, and sulfonate groups that significantly enhance surface polarity and moisture retention, even though the overall rheological properties remain largely unchanged [30,34]. These degradation mechanisms driven by the surface illustrate that sulfur can serve as both a reinforcing agent and a photo-reactive element, highlighting the necessity of stabilization strategies for ensuring long-term durability.

Biochar has been recognized as a potent carbon-rich material that can alleviate both UV- and oxidation-induced deterioration. Its porous structure and presence of heteroatoms enable biochar to decrease chemical aging metrics, hinder the spread of free radicals, and postpone UV-induced hardening in both pure and rubberized binders [35,36,37,38]. In systems containing sulfur, these mechanisms play a crucial role as they effectively counteract photo-oxidative reactions driven by sulfur [39]. Research at the mixture level also demonstrates that biochar improves cracking resistance and rutting performance while minimizing color fading and oxidative surface degradation when exposed to solar radiation [37]. Furthermore, biochar has the ability to adsorb light fractions and volatile organic compounds (VOCs), which aids in stabilizing the colloidal balance of binders and reducing emissions during the aging process [40,41,42,43]. These versatile characteristics position biochar as an attractive option for incorporation into sulfur–bio–oil polymer networks.

Crucially, the radical-scavenging and adsorption properties of biochar directly oppose the sulfur-driven photochemical processes highlighted in recent studies on surface aging, where ultraviolet exposure speeds up the creation of highly hydrophilic sulfur-oxygen compounds [28,40]. By inhibiting these reactions, biochar can act as a stabilizing element that aids in maintaining hydrophobicity and moisture resistance in systems rich in sulfur.

Although there has been extensive research into WCO rejuvenators, sulfur-based polymers, and biochar, previous studies have primarily focused on these modifiers either separately or at the binder level. Investigations into high-RAP binders indicate promising enhancements when utilizing WCO or hybrid systems of WCO and crumb rubber; however, concerns persist regarding their resistance to rutting and their performance over long-term aging [8,9,44,45,46]. Sulfur–bio-oil polymers show significant cross-linking characteristics [24,25,28,29], while biochar consistently enhances photochemical durability and oxidative stability [46,47]. Nevertheless, to the authors’ best knowledge, no research has yet analyzed a combined ternary system made up of sulfur polymer, WCO, TiO_2_ catalyst, and biochar at the mixture level, nor has it been assessed for performance under thermal aging and UV exposure conditions that mimic real-world service.

Although sulfur, waste cooking oil (WCO), and biochar have each been extensively studied as individual modifiers, and their effects on asphalt behavior are well documented, few studies have examined their combined use as an integrated ternary system. The present study, therefore, focuses on the ternary modifier as a sulfur-based polymer formed through TiO_2_-assisted inverse vulcanization and stabilized by biochar. Evaluating the individual components in isolation would not provide meaningful insight in this context, as the observed mechanical and aging-related behavior arises from synergistic interactions within the complete polymer network rather than from the action of any single constituent.

Chemical characterization of sulfur-based polymer network formation via TiO_2_-catalyzed inverse vulcanization has been extensively reported in prior studies [24,26,33]. Building on this established foundation, the present study employs the same catalyst and synthesis methodology to promote sulfur polymerization within the ternary sulfur–waste cooking oil–biochar system. Accordingly, this work focuses on evaluating the mechanical and rheological performance of the resulting materials at the mixture scale, while detailed chemical and mechanistic analyses have been addressed elsewhere.

Given the demonstrated susceptibility of sulfur-rich binders to UV-induced polarity shifts and moisture uptake, evaluating such a ternary system is particularly important for determining whether TiO_2_-assisted sulfur polymerization and biochar stabilization can jointly overcome these degradation pathways. To address this gap, the present study develops and evaluates a low-carbon ternary polymer modifier synthesized from sulfur, WCO, and biochar, with TiO_2_ included to catalyze sulfur network formation. The system is introduced at 10% of binder mass into hot-mix asphalt containing 25% RAP and subjected to coordinated loose-mixture thermal aging and mixture-level UV exposure. Mechanical performance is assessed using the Indirect Tensile Asphalt Cracking Test (IDEAL-CT) and Hamburg Wheel Tracking Test (HWT) to determine whether the ternary polymer system can enhance cracking resistance without compromising rutting performance across aging conditions typical of field environments.

## 2. Materials and Methods

### 2.1. Materials

**Figure 1:**
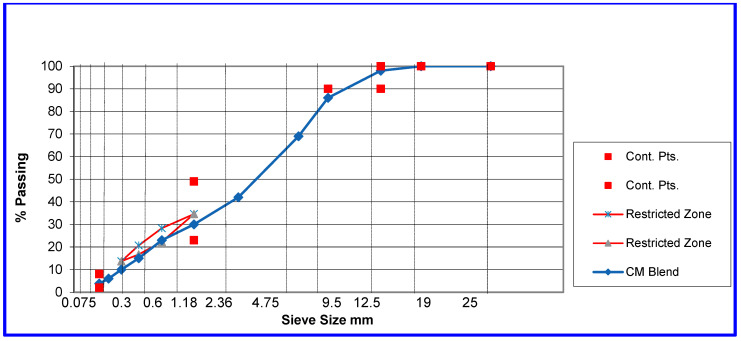
Aggregate grading.

All mixtures used PG 58-16 binder supplied by San Joaquin Refining (Table 1) and aggregates from Vulcan Materials (Corona Drum Plant) meeting the ¾″ hot-mix asphalt (HMA) Type A gradation used locally (Figure 1). A fixed 25% RAP was selected based on commonly adopted agency guidance, consistent with California local agency practices and Caltrans-aligned specifications that allow RAP usage up to approximately 25% in dense-graded HMA mixtures [48].

The approved job mix formula and plant-verified binder contents were 4.7% for the control mix and 10% binder-modified mixtures. Compacted specimens targeted 7.0 ± 0.5% air voids, verified by standard volumetrics [49,50,51].

### 2.2. Binder-Modifier Preparation

The low-carbon ternary modifier was dosed at 10% by binder weight using a 2:4:1 mass ratio of sulfur, waste cooking oil (WCO), and biochar. This ratio was selected based on considerations of chemical reactivity, processability, and prior experimental evidence [24]. Specifically, the sulfur-to-WCO ratio was chosen to provide sufficient unsaturated functionalities from WCO to stabilize polymerized sulfur chains while minimizing free sulfur crystallization. The selected biochar content was informed by literature demonstrating that biochar incorporation in asphalt and sulfur polymer systems promotes interfacial interactions and enhances structural connectivity within polymeric networks. At this dosage, biochar contributes to network reinforcement without impeding polymerization or compromising material homogeneity.

Based on this formulation, elemental sulfur was heated to 160 °C and blended with biochar at a 2:1 sulfur-to-biochar mass ratio for 30 min at the same temperature. The sulfur–biochar mixture was then sheared into the base binder together with WCO at 400 rpm for 18 min. Titanium dioxide (TiO_2_) was added at 0.08% of the sulfur mass to catalyze sulfur-polymer inverse vulcanization and to enhance the photostability of the resulting polysulfide network [24,25]. The modified binder was subsequently held at 160 °C for 60 min to allow completion of network formation prior to mixing with aggregates.

### 2.3. Mixture Production and Short-Term Conditioning

Aggregates were preheated to 160 °C prior to mixing. Loose mixtures were short-term conditioned at 135 ± 3 °C for 2 h per AASHTO R 30 [13]. Gyratory specimens (Ø 150 mm) for IDEAL-CT (62 mm thick) and HWT (60 mm thick) were compacted at 131–135 °C, 600 kPa pressure, 1.16° angle with 5 min squaring time per AASHTO T 312 [49]. Volumetric properties were verified per AASHTO specifications [49,50,51].

### 2.4. Long-Term Thermal and UV Aging

Long-term thermal aging was conducted following the loose-mixture protocol outlined in NCHRP Report 973: 3 and 7 days at 95 °C, which correlates to approximately 3–5 and 12–15 years of field aging at a depth of about 6 mm under the climatic conditions of Southern California [52]. To assess photo-oxidative damage at the mixture level, a laboratory ultraviolet (UV) aging protocol was utilized following 3 days of thermal aging. The specimens underwent 72 h of exposure at an irradiance of 13.6 W/m^2^, across a wavelength range of 315–400 nm, with a distance of 15–18 cm between the loose mixture and the lamp. The temperature in the chamber was kept at roughly 40 °C, resulting in surface temperatures of the loose mixture between 50 and 55 °C at approximately 27% relative humidity.

The specified wavelength range and irradiance are in line with the UVA–near-UV spectrum typically used in laboratory analyses to assess photo-oxidative aging in asphalt materials, while the lamp distance was calibrated to ensure consistent irradiance across the specimen surface [53]. This UV protocol was adapted from mixture-level procedures previously reported for examining the protective effects of biochar and other modifiers in asphalt systems [35,36]. The UV conditioning applied aimed to provide a comparative analysis of photo-oxidative aging under controlled laboratory settings rather than replicating field exposure through direct radiation equivalent simulation.

In total, four aging conditions were established for both the control and modified mixtures (with 10% modifier): without aging (WA), 3 days of thermal aging (3 d), 7 days of thermal aging (7 d), and a combined aging process involving 3 days of thermal aging followed by 3 days of UV exposure (UV).

### 2.5. Mechanical Testing

**Figure 2:**
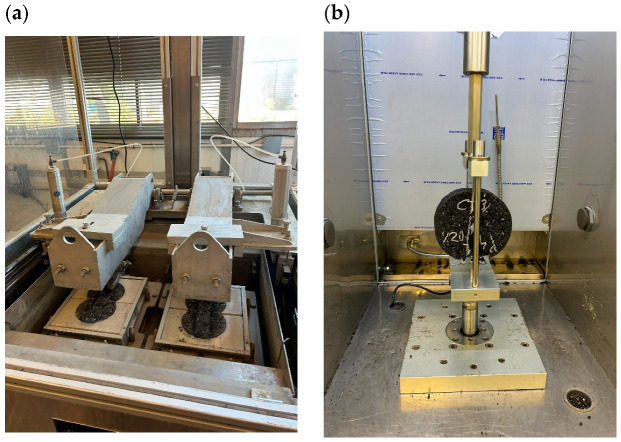
(**a**) Hamburg wheel track test setup; (**b**) IDEAL-CT Test Setup.

The measurement of crack resistance was conducted using the IDEAL-CT (ASTM D8225) at an intermediate temperature of 25 °C, a loading rate of 50 mm/min, and a termination load of 0.1 kN (as shown in Figure 2). The results were determined based on the peak load, the post-peak slope at 75% of the peak, and the fracture energy; three replicate specimens were tested for each condition [54].

Rutting (illustrated in Figure 2) and testing for moisture susceptibility were performed on four replicate specimens using the Hamburg Wheel Tracking Test (HWT) at 50 °C in water until reaching 20,000 passes, following the guidelines of the AASHTO T 324 test and Caltrans procedure [54]. For analysis, the primary staging benchmark would be set at 15,000 passes.

## 3. Results and Discussion

The IDEAL-CT test was performed at a temperature of 25 °C to evaluate cracking resistance using the Cracking Tolerance Index (${CT}_{Index}$), following ASTM D8225 guidelines. The HWT test was conducted in water at 50 °C for 20,000 cycles, according to AASHTO T 324, and based on Caltrans standards, rutting performance was evaluated after 15,000 passes.

### 3.1. Indirect Tensile Asphalt Cracking Test

**Figure 3:**
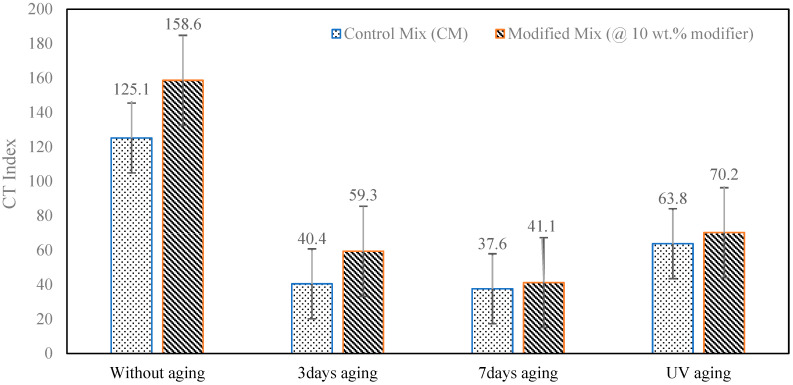
IDEAL-CT results of aged and unaged specimens of the control mix and the modified mix.

The cracking resistance of both control and modified asphalt mixtures was assessed using the Indirect Tensile Asphalt Cracking Test (IDEAL-CT), with results displayed in Figure 3 for various aging conditions. In most scenarios, the introduction of a ternary modifier consisting of sulfur, waste cooking oil, and biochar at a 10 wt% binder replacement improved the ${CT}_{Index}$ values, which suggests a better resistance to crack initiation and early crack development.

In unaged conditions, the modified mixture had a ${CT}_{Index}$ of 158.6, compared to 125.1 for the control mixture, representing about a 27% increase. This enhancement indicates that the sulfur-based modification boosts fracture tolerance before aging, allowing the mixture to absorb more energy during crack formation, which aligns with findings from previous research on sulfur polymer-modified binders [24,25].

After 3 days of thermal aging, both mixtures showed significant drops in ${CT}_{Index}$ due to stiffening from aging; however, the performance disparity widened, with the control mixture at 40.4 and the modified mixture retaining a ${CT}_{Index}$ of 59.3, reflecting a 47% improvement over the control. Statistical analysis (Table 2) confirmed that this difference was significant at the 10% level (*p* = 0.034), with even stronger evidence under a one-tailed test (*p* = 0.017). This suggests that the modified mixture ages more slowly and is less prone to early oxidative hardening during its initial service life.

After 7 days of thermal aging, ${CT}_{Index}$ values further decreased to 37.6 for the control and 41.1 for the modified mixture. Although the modified mixture maintained a higher ${CT}_{Index}$ (about 9% more), the difference was not statistically significant, indicating that prolonged thermal aging reduces the relative advantages of the modifier as oxidative hardening becomes the primary factor affecting cracking behavior.

When subjected to combined thermal and ultraviolet (UV) aging—consisting of 3 days of thermal conditioning followed by UV exposure—the modified mixture showed better cracking resistance once again. The ${CT}_{Index}$ values recorded were 70.2 for the modified and 63.8 for the control, reflecting around a 10% improvement. This suggests that the ternary modifier offers some protection against photo-oxidative degradation.

**Figure 4:**
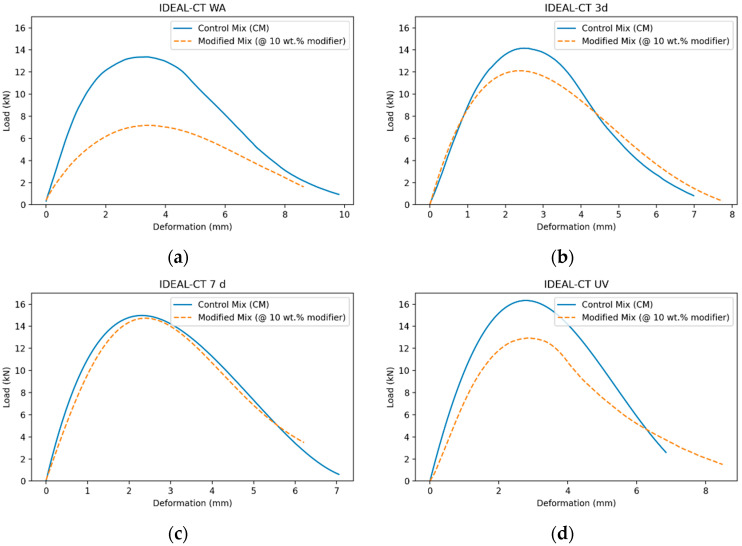
(**a**) IDEAL-CT load–deformation curve for unaged specimens (WA); (**b**) IDEAL-CT load–deformation curve after 3 days of thermal aging; (**c**) IDEAL-CT load–deformation curve after 7 days of thermal aging; (**d**) IDEAL-CT load–deformation curve after combined UV and thermal aging.

The load–displacement responses illustrated in Figure 4 shed light on the ${CT}_{Index}$ trends. In the IDEAL-CT framework, the ${CT}_{Index}$ results depend not only on the peak load but also on the post-peak softening behavior, as illustrated by the slope of the descending part of the load–displacement curve. For both 3-day and 7-day thermally aged conditions, the modified mixture showed a broader post-peak response, demonstrating a greater displacement capacity for similar load levels. This indicates a more gradual softening response after peak load, contributing to higher ${CT}_{Index}$ values, even with comparable or lower peak loads. The modified mixture similarly exhibited broader post-peak tails under combined thermal and UV aging compared to the control mixture. This behavior suggests that the differences in ${CT}_{Index}$ are more closely linked to post-peak deformation characteristics rather than just peak load, in line with the IDEAL-CT fracture characterization framework [55].

The observed improvements in cracking resistance stem from the synergistic effects of the ternary modifier components. Waste cooking oil rejuvenates the mixture by restoring maltene fractions and reducing stiffness from aging, while biochar helps stabilize the binder’s colloidal structure and limits oxidative hardening [7,8,24,25,40]. Furthermore, biochar’s ability to block UV and scavenge free radicals, combined with the photo-stabilizing function of sulfur polymer networks and TiO_2_, helps slow UV-induced degradation [24,35,36,56]. Collectively, these mechanisms enhance post-peak deformation capacity and fracture energy, potentially leading to better cracking resistance during the early service life of asphalt pavements.

### 3.2. Hamburg Wheel Tracking Test

**Figure 5:**
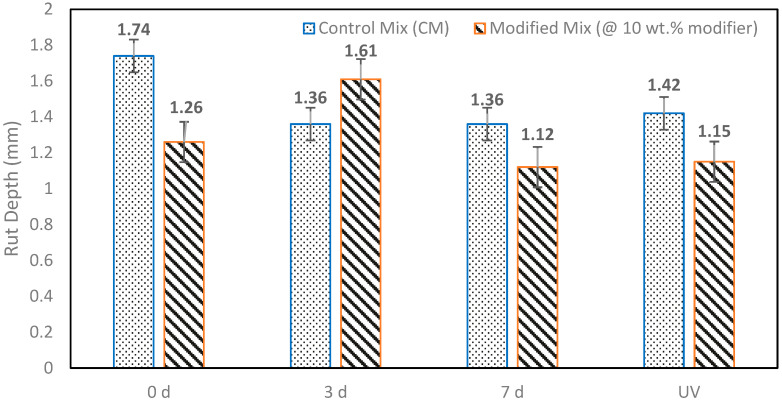
Rut depth at 15,000 loading cycles for aged and unaged specimens of the control mix and the modified mix.

Figure 5 displays the rut depths recorded after 15,000 wheel passes for both the control and modified mixtures under various aging conditions. In general, all mixtures demonstrated relatively low levels of permanent deformation, indicating sufficient resistance to rutting, regardless of the aging status.

In unaged conditions, the modified mixture recorded a rut depth of 1.26 mm, whereas the control mixture measured 1.74 mm, reflecting a reduction of about 28% in rut depth. This enhancement suggests that the inclusion of the sulfur–waste cooking oil–biochar ternary modifier improves resistance to early-stage permanent deformation, even in the presence of waste cooking oil, which typically leads to binder softening.

After 3 days of thermal aging, the control mixture showed a rut depth of 1.36 mm, while the modified mixture had a rut depth of 1.61 mm, indicating an approximate 18% increase in rutting for the modified mixture compared to the control. This behavior is ascribed to the short-term softening effect of waste cooking oil, which may temporarily diminish mixture stiffness prior to adequate oxidative hardening and sulfur-based cross-linking.

Following 7 days of thermal aging, the modified mixture once again exhibited better performance than the control mixture, with rut depths of 1.12 mm and 1.36 mm, respectively. This translates to an approximately 18% decrease in rut depth for the modified mixture. This trend suggests that extended aging facilitates further stabilization of the modified binder system, countering the initial softening caused by waste cooking oil and enhancing resistance to permanent deformation through the development of a sulfur polymer network and biochar reinforcement.

Under simultaneous thermal and ultraviolet (UV) aging, the modified mixture displayed the lowest rut depth across all conditions at 1.15 mm, compared to 1.42 mm for the control mixture, corresponding to an approximate 19% decrease in rutting. This superior performance under UV exposure suggests that the ternary modifier affords additional protection against photo-oxidative degradation. The combined effects of sulfur polymer cross-linking, biochar UV shielding, and TiO_2_-mediated stabilization likely contribute to preserving mixture stiffness without causing brittleness.

**Figure 6:**
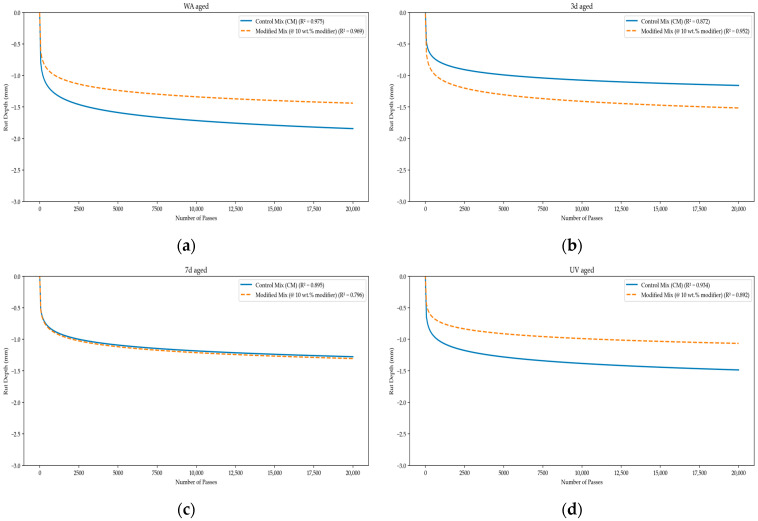
(**a**) Rut depth versus number of wheel passes for unaged (WA) specimens; (**b**) rut depth versus number of passes after 3 days of thermal aging; (**c**) rut depth versus number of passes after 7 days of thermal aging; (**d**) rut depth versus number of passes after combined UV and thermal aging.

Figure 6 demonstrates the evolution of rut depth with wheel passes for both mixtures across all aging conditions. The rutting curves showed smooth, continuous accumulation without inflection points or breaks in slope, indicating no signs of moisture-induced damage or stripping. The resemblance in rutting slopes between the control and modified mixtures further verifies that the ternary modifier does not negatively impact high-temperature deformation behavior.

At the Caltrans reference point of 15,000 passes, no statistically significant differences in rutting performance were identified between the control and modified mixtures across aging conditions, despite variations in average rut depth values. Average rut depths consistently fell between 0.8 and 1.8 mm, which is significantly below the 12.5 mm failure threshold set by Caltrans. These findings illustrate that the sulfur–waste cooking oil–biochar modifier does not introduce penalties related to rutting, even after prolonged aging and UV exposure [7,25,28].

From a performance-balance viewpoint, the rutting results hold particular importance when evaluated alongside the notable improvements seen in cracking resistance. The ternary modifier enhances both early-life and aged cracking performance while maintaining similar or improved rutting resistance, suggesting a reduction in the typical trade-off between cracking and rutting within the constraints of the experimental dataset [28,36]. This balance reinforces the practical suitability of the modifier in RAP-rich asphalt mixtures, allowing for enhanced durability and sustainability without sacrificing high-temperature performance.

## 4. Environmental Impact Assessment

The environmental impacts of the low-carbon ternary-modified binder were assessed using a simplified GWP calculation method that accounts for bitumen displacement and the carbon effects of each component [36]. The cradle-to-gate GWP range reported in NAPA’s Carbon Footprint of Asphalt Pavements decarbonization assessment (SIP-109, 2024) was used, and the average value of 484 kg CO_2_-eq per ton was adopted in this study [57,58]. This value also aligns with industry-wide carbon-footprint datasets [59], which identify asphalt binder production as one of the dominant contributors to A1–A3 emissions. Given this high baseline, even partial displacement of petroleum binder can yield meaningful reductions in embodied carbon.

In this system, 10 wt% of the binder is replaced by a ternary sulfur–WCO–biochar modifier following a 2:4:1 mass ratio. Sulfur and waste cooking oil (WCO) were assigned negligible GWP burdens, consistent with their classification as refinery by-products and post-consumer waste streams, respectively. Their inclusion therefore contributes entirely through bitumen displacement credit, which in this case reduces the GWP by approximately 13.8 kg CO_2_-eq (sulfur) and 27.6 kg CO_2_-eq (WCO) per ton of binder (Table 3). The biochar fraction provides two environmental benefits: a bitumen displacement credit of 6.9 kg CO_2_-eq and an additional net-negative emission of 5.9 kg CO_2_-eq, consistent with reported sequestration values of approximately −415.6 kg CO_2_-eq per ton of biochar [60,61].

The only component with a positive environmental burden is the TiO_2_ catalyst. At a dosage of 0.002 wt%, its contribution is small, adding only 0.15 kg CO_2_-eq per ton of binder, based on a carbon intensity of 7.47 kg CO_2_-eq per kilogram [62].

When all mass-weighted contributions are combined, the modified binder exhibits a net GWP of approximately 430 kg CO_2_-eq per ton, compared to 484 kg CO_2_-eq for the neat binder. This corresponds to an 11.2% reduction in global warming potential, originating from (i) substitution of high-GWP petroleum binder with waste-derived components, and (ii) the carbon-negative behavior of biochar. These results reinforce findings from previous studies showing that sulfur polymer and biochar systems can reduce environmental burdens by nearly 11% while enhancing the long-term stability and performance of modified asphalt binders.

## 5. Conclusions

This research explored a low-carbon, sulfur-based ternary polymer modifier created from elemental sulfur, waste cooking oil, and metal-rich biochar through TiO_2_-catalyzed inverse vulcanization and assessed its effectiveness at the asphalt mixture level with reclaimed asphalt pavement (RAP) included. The sulfur polymer was added at 10% of the binder weight into mixtures containing 25% RAP and was evaluated under unaged conditions, thermal aging, and a combination of thermal and ultraviolet aging.

The IDEAL-CT results revealed improved cracking resistance for the modified mixture under unaged conditions and after 3 days of thermal aging, with diminishing differences noted after extended thermal aging. After 7 days of thermal aging, the modified mixture exhibited slightly higher ${CT}_{Index}$ values than the control, though the differences were not statistically significant. Under conditions of combined thermal and ultraviolet aging, modest enhancements in ${CT}_{Index}$ were noted, indicating slight resistance to photo-oxidative degradation mechanisms.

Hamburg Wheel Tracking results showed that the integration of the sulfur polymer did not negatively impact rutting or moisture performance across all aging scenarios. Although a temporary increase in rut depth was detected after 3 days of thermal aging, rutting resistance during prolonged thermal aging and combined thermal–ultraviolet aging was comparable to or improved compared to the control mixture. In all instances, rut depths remained significantly below the Caltrans failure threshold, and there was no evidence of moisture-induced damage.

A basic environmental assessment indicated that substituting 10 wt% of petroleum binder with the sulfur polymer decreased the cradle-to-gate global warming potential from 484 to about 430 kg CO_2_-eq per ton of binder, reflecting an approximate 11% reduction. This decline was primarily due to replacing virgin binder with waste-derived materials and the carbon-negative impact of biochar.

In conclusion, the study results show that the proposed sulfur polymer system can enhance cracking resistance even when exposed to select accelerated aging conditions without compromising resistance to rutting or moisture, while also lowering embodied carbon in asphalt mixtures. These findings underscore the potential of sulfur-based polymer networks as a viable low-carbon modification in asphalt pavements that incorporate reclaimed materials. Further research should study surface aging behavior and sulfur polymer dosage sensitivity to complement the performance evaluation and refine mixture design.

## Figures and Tables

**Table 1:** Asphalt Binder Properties.

Property	Test Method	Results (PG 58-16)	Spec Limit
Binder Identification and Composition			
Asphalt Binder Grade	AASHTO M320	PG 58-16	PG 58-16
Asphalt Binder Content (%)	AASHTO T308	4.70%	4.5–5.2% (typical)
RAP Binder Content (%)	ASTM D2172	4.77%	N/A
Original Binder Properties			
Flash Point (°C)	ASTM D92/AASHTO T48	302	≥230
Viscosity @ 135 °C (Pa·s)	ASTM D4402/AASHTO T316	0.25	≤3.0
G*/sin(δ) @ 58 °C (kPa)	AASHTO T315	1.74	≥1.00
Phase Angle, δ (°)	AASHTO T315	88.8	–
RTFO Aged Binder Properties			
Mass Loss (%)	AASHTO T240	−0.08	≤1.00
G*/sin(δ) @ 58 °C (kPa)	AASHTO T315	3.5	≥2.20
Phase Angle, δ (°)	AASHTO T315	87.4	–
PAV Aged Binder Properties			
G*·sin(δ) @ 25 °C (kPa)	AASHTO T315	4300	≤5000
Phase Angle, δ (°)	AASHTO T315	57.3	≥42°
BBR Test @ −6 °C	AASHTO T313		
Stiffness, S (MPa)	AASHTO T313	77.5	≤300
m-value	AASHTO T313	0.4	≥0.300
Other Properties			
Specific Gravity @ 60 °F	ASTM D70	1.0154	–

**Table 2:** Summary of statistical analysis of CT_index_ between control and modified mixtures.

${CT}_{index}$	Levene’s Test for Equality of Variances		*t*-test for Equality of Means							
	F	Sig.	t	df	Significance		Mean Difference	Std. Error Difference	95% Confidence Interval of the Difference	
					One-Sided *p*	Two-Sided *p*			Lower	Upper
	0.11	0.761	−3.17	4	0.017	0.034	−18.9	5.98	−35.5	−2.3

**Table 3:** Global warming potential assessment of modified asphalt binder.

Component/Effect	wt% of Binder	Effect Type	ΔGWP (kg CO_2_-eq/ton Binder)
Baseline binder (unmodified)	100	Reference GWP	+484.0
Sulfur—binder displacement	2.86	Displacement credit (bitumen)	−13.8
WCO—binder displacement	5.71	Displacement credit (bitumen)	−27.6
Biochar—binder displacement	1.43	Displacement credit (bitumen)	−6.9
Biochar—net carbon sequestration	1.43	Negative emission (sequestration)	−5.9
TiO_2_	0.002	Additional positive GWP	+0.15
Net GWP of modified binder			429.8
Percent reduction vs. baseline			11.2%

## Data Availability

The original contributions presented in this study are included in the article. Further inquiries can be directed to the corresponding authors.

## Abbreviations

The following abbreviations are used in this manuscript:

HWT	Hamburg Wheel Tracking Test
RAP	Reclaimed Asphalt Pavement
UV	Ultraviolet
IDEAL-CT	Indirect Tensile Asphalt Cracking Test
WA	Without Aging
NCHRP	National Cooperative Highway Research Program
HMA	Hot Mix Asphalt
